# Effective treatment of refractory aplastic anemia with romiplostim after failure of multiple thrombopoietin receptor agonists: a single-center retrospective study

**DOI:** 10.1080/07853890.2025.2514791

**Published:** 2025-06-19

**Authors:** Xijuan Lin, Chen Yang, Ziwei Liu, Junling Zhuang, Miao Chen, Bing Han

**Affiliations:** a Department of Hematology, Peking Union Medical College Hospital, Chinese Academy of Medical Science, Beijing, China; b Eight-year Medical Doctor Program, Peking Union Medical College Hospital, Chinese Academy of Medical Science, Beijing, China

**Keywords:** Aplastic anemia, refractory, romiplostim, efficacy, safety

## Abstract

**Background:**

Romiplostim (ROM), a second-generation thrombopoietin receptor agonist (TPO-RA), has shown promising results in patients with refractory aplastic anemia (AA); however, its optimal dosage, efficacy, and safety in patients with refractory AA who experienced treatment failure with immunosuppressive therapy (IST) and other types of TPO-RAs remain unclear. In the present study, we aimed to retrospectively assess the efficacy and safety of high-dose ROM in these patients.

**Patients and methods:**

Patients who received ROM consecutively for at least 3 months between 8 April 2023 and 23 October 2023, and were followed up for at least 6 months after therapy were analyzed. All enrolled patients had failed to respond and stopped the previous treatments for at least 3 months before receiving ROM.

**Results:**

Eleven patients were followed up for at least 6 months; all experienced treatment failure with IST and at least two types of other TPO-RAs. They had a median age of 54 years (range: 27–77 years), and eight (72.7%) were women. The patients’ initial and maximal ROM dose was 20 μg/kg per week. At a median follow-up of 8 months (range, 6–8 months), 72.7% (8/11) of the patients reached the response criteria at a median of 1 month (range: 1–3 months). Of these patients, 54.5% (6/11) met the criteria for a complete response at a median of 2.5 months (range: 1–3 months), and 27.3% (3/11) achieved a trilineage response. No severe ROM-related adverse events that led to treatment discontinuation or dosage reduction were observed. Notably, 12.5% (1/8) of the responders relapsed at 5 months after stopping ROM for 2 months.

**Conclusion:**

High-dose ROM with an initial dosage of 20 μg/kg per week is effective and safe for patients with refractory AA who experienced treatment failure with IST and multiple oral TPO-RAs, exerting a rapid response.

## KEY MESSAGES

Romiplostim was safe and effective at an initial dosage of 20 μg/kg per week, with rapid responses in patients with refractory aplastic anemia who experienced treatment failure with immunosuppressive therapy and multiple oral thrombopoietin receptor agonists.

High-dose romiplostim can be recommended as salvage treatment for patients with refractory aplastic anemia.

## Introduction

Aplastic anemia (AA) is a rare hematological disease characterized by pancytopenia and hypocellular bone marrow [1]. Immunosuppressive therapy (IST), which includes anti-thymocyte globulin (ATG) and cyclosporine (CsA) and the probable inclusion of eltrombopag (EPAG), is the preferred first-line treatment for patients with severe AA (SAA) without compatible sibling donors. IST yields a response in 60–70% of cases [2–4]. However, despite the relatively higher overall response rate (ORR) in patients with non-SAA (NSAA), approximately 25% of patients with NSAA fail to respond to ATG plus CsA, and 54% fail to respond to CsA monotherapy [5].

Therefore, the treatment of patients with refractory AA who fail to respond to IST is a formidable challenge. Before the introduction of thrombopoietin receptor agonists (TPO-RAs), other therapeutic options, such as danazol, cyclophosphamide, and alemtuzumab, often failed to elicit effective outcomes in patients with refractory AA ineligible for hematopoietic stem cell transplantation (HSCT). Moreover, these alternatives are associated with a considerable risk of infectious complications [6–8]. EPAG, a type of TPO-RA, is the most widely used and highly recommended alternative treatment; however, it only induces a response in approximately 20–50% of patients with refractory AA [9–11]. Furthermore, the efficacy and safety of other TPO-RAs, including avatrombopag, hetrombopag, lusutrombopag, and romiplostim (ROM), are still under investigation. Therefore, there is an urgent need for more studies regarding other types of TPO-RAs to provide alternative treatments for patients with refractory AA. The suggested treatments for patients with refractory AA who have failed to respond to one or more types of TPO-RAs are also worth studying.

ROM, a second-generation TPO-RA, is a synthesized F_C_-peptide fusion protein with a mechanism of action similar to that of native TPO. Like other types of TPO-RAs, it attaches to and activates the TPO receptor found on megakaryocytes and hematopoietic stem cells, resulting in potential trilineage responses in patients with AA [12–14]. Studies have demonstrated promising results regarding the efficacy and safety of ROM in patients with refractory AA, with an ORR of 70 to 95% and few severe adverse events (AEs) [15–18]. However, in previous cohorts, only a few lines of treatment, mainly ATG, CsA, granulocyte-colony stimulating factor, and EPAG, have been applied and focused on. The efficacy and safety of ROM in patients who failed to respond to TPO-RAs, except for EPAG, have not been examined. In addition, patients who received ROM at different initial and maximal dosages in previous studies demonstrated significant differences in the ORR. A Japanese cohort with an initial and a median maximal dosage of 10 μg/kg and 20 μg/kg per week, respectively, achieved an ORR of 76% [16] However, a French cohort with an initial dosage of 5 μg/kg per week and a median maximal dosage of 10 μg/kg per week achieved an ORR of 12.5% [19]. Therefore, the optimal initial and maximal doses of ROM are worth investigating.

Thus, in the present study, we retrospectively assessed the efficacy and safety of high-dose ROM with an initial and maximal dosage of 20 μg/kg per week for patients with refractory AA who had failed to respond to IST and at least two other TPO-RAs, to further investigate the efficacy, safety, and optimal dose of ROM in patients with refractory AA.This article is in accordance with our poster presented at the 2024 ASH Annual Meeting. It extends the findings from the poster by providing more detailed information on high-dose ROM for refractory AA. [20].

## Patients and methods

### Patients

Data were collected retrospectively from patients with refractory AA treated with ROM at the Peking Union Medical College Hospital between April 2023 and October 2023. Refractory AA was defined as the absence of a response to previous treatments including IST and previous TPO-RAs. The inclusion criteria were: (1) age ≥18 years; (2) refractory AA diagnosis and meeting at least one of the following criteria before ROM: hemoglobin concentration <90 g/L, platelet count <30 × 10^9^/L, or neutrophil count <0.5 × 10^9^/L; (3) not receiving HSCT or being HSCT candidates; (4) treatment with ROM should be continued for at least 3 months if there was no response and had been followed up for at least 6 months after ROM; (5) ROM as the only treatment during the study.

Patients finally diagnosed with congenital AA (Fanconi anemia and congenital dyskeratosis), those who had paroxysmal nocturnal hemoglobinuria (PNH) granulocyte clone size ≥ 50% determined using flow cytometry, those who had evidence of clonal hematologic bone marrow disorders on cytogenetics, or those who had concomitant malignancies before ROM were excluded from the final analysis.

SAA was defined as having marrow cellularity of <25% (or 25–50% with <30% residual hematopoietic cells) and at least two of the following: neutrophil count <0.5 × 10^9^/L, platelet count <20 × 10^9^/L, or reticulocyte count <60 × 10^9^/L; very SAA (VSAA) was defined as having SAA plus neutrophils <0.2 × 10^9^/L; NSAA was defined as AA that not fulfill the criteria for SAA or VSAA [1]; transfusion-dependent AA was defined as AA with transfusions in the 8 weeks before the first ROM dose [15]. Refractory AA was identified when the patient showed no response after > 6 months of CsA in combination with at least two full-dose TPO-RAs (except ROM) for at least 3 months. To exclude the impact of previous treatments, patients had stopped the previous IST and TPO-RAs for at least 3 months before receiving ROM.

Before data collection, all patients gave written informed consent. The study was approved by the Ethics Committee of Peking Union Medical College Hospital (Reference No. I-25PJ0248) and was conducted according to the Declaration of Helsinki.

### Treatment regimens

Patients were treated with ROM for at least 3 months if they did not respond. The initial dose of ROM was 20 μg/kg per week; the ROM dose could be tapered or stopped if the platelet count increased to ≥200 × 10^9^/L without transfusion or at the patient’s wish. Other treatments included transfusion if hemoglobin was < 60 g/L, platelet count was < 30 × 10^9^/L. The dose of granulocyte-colony stimulating factor was 5 μg/kg/day if the absolute neutrophil count was < 0.5 × 10^9^/L.

Clinical characteristics, including age, sex, severity, previous treatment, time since ROM diagnosis, transfusion requirement, ROM doses, and critical laboratory hematological parameters were collected at baseline and at different time points after ROM treatment. Response to ROM was assessed at 3 and 6 months and at the end of follow-up after the first ROM dose.

### Assessment of response

The hematological response was defined according to the guidelines for AA. Complete response (CR) was defined as having a platelet count >150 × 10^9^/L, hemoglobin >120 g/L for men (110 g/L for women), and an absolute neutrophil count > 1.5 × 10^9^/L. Partial response (PR) was defined as having at least one line of hematological response. Platelet response was defined as an increase of at least 20 × 10^9^/L above the baseline platelet level (pretreatment platelet level of < 20 × 10^9^/L), platelets being restored to normal or doubled counts compared with those at baseline, or platelet transfusion independence in previously transfusion-dependent patients. An erythroid response was defined as an increase in the hemoglobin level of at least 30 g/L (pretreatment hemoglobin level of < 60 g/L), hemoglobin being restored to normal or doubled values compared with those at baseline, or red blood cell transfusion independence in previously transfusion-dependent patients. A neutrophil response was defined as an increase in the absolute neutrophil count of at least 0.5 × 10^9^/L (pretreatment absolute neutrophil count of < 0.5 × 10^9^/L) or absolute neutrophil count restored to normal or doubled values compared with those at baseline. No response was defined as the absence of any response[1]. The overall response (OR) was defined as achieving PR or CR. Relapse was defined as a substantial or progressive decline in at least one blood lineage count of responders requiring reinitiation or augmentation of ROM.

### Assessment of AEs and clone evolution

AEs were acquired from medical records and telephone interviews and were graded following the Common Toxicity Criteria of the National Cancer Institute version 5.0. Follow-up PNH clonal evolution and other clone evolution tests, including karyotype and gene analyses, were also documented. Next-generation sequencing was performed on peripheral blood to identify genetic mutations associated with myelodysplastic syndromes. The percentage of flaer negative granulocytes in peripheral blood measured by flow cytometry was documented as the size of PNH clone. Chromosome karyotype analysis was performed using bone marrow samples.

### Statistical analysis

All data were analyzed and visualized using Prism 8.0.2 (GraphPad Software Inc., San Diego, CA, USA). Continuous data were presented as medians and ranges. Categorical variables were presented as frequencies or ratios (%). Critical laboratory parameters were analyzed using t-tests. All analyses were two-sided, with statistical significance set at *p* < 0.05.

## Results

### Patients

Nineteen patients were treated with ROM between 8 April 2023, and 23 October 2023. Four patients were lost to follow-up within 6 months after ROM, and four switched to other therapies within 2 months due to economic reasons. Finally, 11 patients met the inclusion criteria and were analyzed. They had a median age of 54 years (range: 27–77 years), and eight (72.7%) were women. Among them, one (9.1%) had VSAA, one (9.1%) had SAA, and nine (81.8%) had TD-NSAA. None of the patients had positive PNH clones or abnormal karyotypes at baseline. However, two (18.2%) had *ASXL1* mutation, with mutant allele frequencies of 2.6% and 1.5% at baseline and 2.1% and 0.8% at the end of follow-up.

Before ROM, all enrolled patients had been treated with CsA (3–5 mg/kg) for at least 6 months with no response. Other immunosuppressive therapies included androgens, tacrolimus, ATG, and sirolimus in seven (63.6%), seven (63.6%), one (9.1%), and one (9.1%) patients, respectively. These patients had been treated with at least two other TPO-RAs with no response before ROM, including 7/11 (63.6%) treated with EPAG at a median dosage of 100 mg (range: 50–150 mg) once daily for 6 months (range: 3–15 months), 4/11 (36.4%) with hetrombopag at a median dosage of 10.0 mg (range: 7.5–15 mg) once daily for 6 months (range: 3–10 months), and all patents (11/11; 100%) with avatrombopag 60 mg once daily for 5 months (range: 3–12 months). All patients were transfusion-dependent; their baseline characteristics are presented in Table 1.

**Table 1.** Baseline characteristics of patients enrolled.

Characteristics	Patients (*N* = 11)
Age at ROM start (years)	54 (27–77)
Gender	
Male—no. (%)	3 (27.3)
Time from diagnosis to ROM (years)	5 (1–36)
Duration without previous TPO-RAs and IST before ROM (months)	4 (3–9)
Severity—no. (%)	
VSAA	1 (9.1)
SAA	1 (9.1)
NSAA	9 (81.8)
Previous treatment—no. (%)	
CsA	11 (100)
Eltrombopag	7 (63.6)
Hetrombopag	6 (54.5)
Avatrombopag	11 (100)
Androgen	7 (63.6)
Tacrolimus	7 (63.6)
ATG	1 (9.1)
Sirolimus	1 (9.1)
With PNH clone, *N* (%)	0 (0.0)
With abnormal karyotype or MDS-related gene mutations, *N* (%)	2 (18.2)
Comorbidity, No. (%)	
Pulmonary embolism	1 (9.1)
Diabetes	1 (9.1)
Hypertension	1 (9.1)
Hepatitis	1 (9.1)
Liver cirrhosis	1 (9.1)
Transfusions required—no. (%)	
Platelets	11 (100)
Packed red cells	7 (63.6)
Laboratory values before ROM	
Platelets (×10^9^ /L)	8 (1–39)
Neutrophils (×10^9^ /L)	2.6 (0.2–5.9)
Hemoglobin (g/L)	56 (42–126)
Lineage number of cytopenia, N (%)	
Cytopenia = 1	4 (36.4)
Thrombocytopenia	4 (36.4)
Cytopenia = 2	3 (27.3)
Cytopenia = 3	4 (36.4)
Serum ferritin/ (ng/mL), median (range)	436 (26–2132)
Follow-up after ROM (months)	6 (6–8)

TPO-RA: Thrombopoietin receptor agonists; IST: Immunosuppressive therapy; ROM: Romiplostim; NSAA: Non-severe aplastic anemia; SAA: Severe aplastic anemia; VSAA: very severe aplastic anemia; ATG: Anti-thymocyte globulin; CsA: Cyclosporin A; PNH: Paroxysmal nocturnal hemoglobinuria.

The patients were treated with ROM for a median of 4 months (range: 3–8 months), and all patients received ROM at an initial dose of 20 μg/kg per week. The median duration of treatment at the maximum ROM dose was 3 months (range: 1–6 months), with median ROM dose of 10 μg/kg per week (range: 0–20 μg/kg per week) at the end of the follow-up. After a median follow-up of 8 months (range: 6–8 months), 4 patients (36.4%) discontinued ROM, while 5 patients (45.5%) had their dosage tapered. One patient (9.1%) discontinued ROM after the platelet count increased to ≥200 × 10^9^/L at 3 months. Two patients (18.2%) reduced their dose to 10 μg/kg per week after the platelet count increased to ≥ 200 × 10^9^/L. Other patients discontinued ROM or tapered the dose due to financial reasons or at their own will.

### Efficacy

At a median follow-up of 8 months (range: 6–8 months), 8 patients (72.7%) and 6 patients (54.5%) had reached OR and CR, respectively. OR was reached at a median of 1 month (range: 1–3 months) and CR at a median of 2.5 months (range: 1–3 months). The OR rates were 72.7% (8/11), 63.6% (7/11), and 63.6% (7/11) at 3 months, 6 months and at the end of follow-up. The CR rates were 45.5% (5/11), 18.2% (2/11), and 18.2% (2/11) at 3 months, 6 months, and at the end of follow-up.

**Figure 1.**
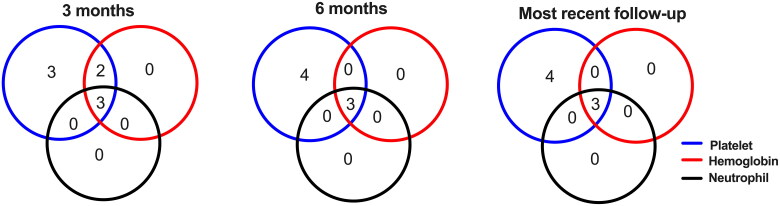
Lineage response to ROM during treatment. The Venn diagrams show the number of patients with unilineage, bilineage, and trilineage hematological response at 3-months (left), 6-months (middle), and the end of follow-up (right) from the initiation of ROM treatment. ROM: romiplostim.

Figure 1 shows the number of patients with unilineage, bilineage, and trilineage hematologic responses at 3 months, 6 months and the end of the follow-up period. At 3 months, three (27.3%), two (18.2%), and three (27.3%) patients had unilineage, bilineage, and trilineage responses, respectively. At 6 months, four (36.4%), zero (0.0%), and three (27.3%) patients had unilineage, bilineage, and trilineage responses, respectively. At the end of follow-up, four (36.4%), zero (0.0%) and three (27.3%) patients had unilineage, bilineage, and trilineage responses, respectively.

**Figure 2.**
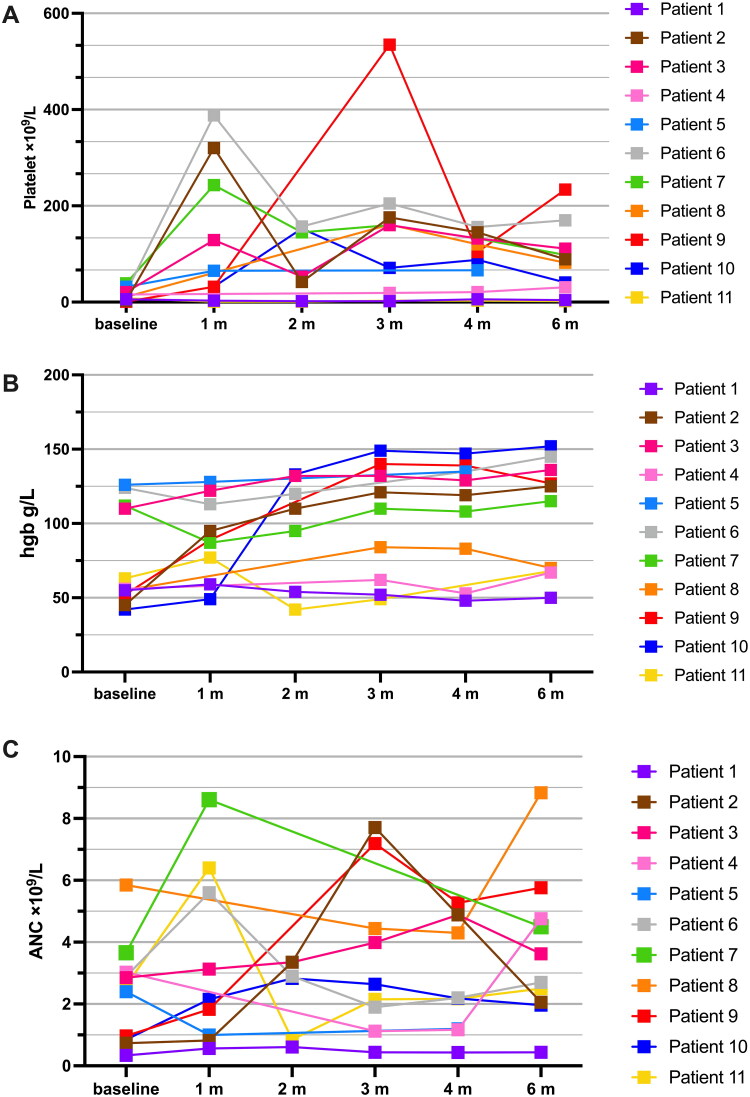
Longitudinal hematologic improvements of patients with refractory AA treated with ROM. Longitudinal blood counts of all 11 patients are shown. (A) Platelet count, (B) hemoglobin concentration, and (C) neutrophil count. ROM, romiplostim; AA, aplastic anemia

During the entire follow-up period, 8 patients (72.7%) of the patients showed a platelet response, with a median increase in platelet count of 55.5 × 10^9^/L (range: 22–233 × 10^9^/L) at the end of follow-up (Figure 2A), and 63.6% (7/11) of the patients became platelet transfusion-independent at a median of 3 months (range: 2–4 months). Erythroid responses were observed in 57.1% (4/7) of the patients (Figure 2B), with a median increase in hemoglobin level of 63.5 g/L (range: 15–110 g/L), and 57.1% (4/7) of the patients became erythrocyte transfusion-independent at a median of 2.5 months (range: 2–3 months). 75% (3/4) of patients had a neutrophil response (Figure 2C), with a median increase of 1.11 × 10^9^/L (range: 0.81–4.79 × 10^9^/L).

The platelet counts increased significantly compared with those at baseline, 3 months, 6 months, and the end of follow-up. Compared with 8 × 10^9^/L (range: 1–39 × 10^9^/L) at baseline, platelet counts were 160 × 10^9^/L (range: 2–535 × 10^9^/L), 89 × 10^9^/L (range: 2–234 × 10^9^/L) and 41 × 10^9^/L (range: 2–234 × 10^9^/L) at 3 months, 6 months and at the end of follow-up, respectively. However, there was no significant increase in hemoglobin level or absolute neutrophil count at 3 months, 6 months, or at the end of follow-up.

### AEs

Notably, most patients tolerated the treatment well, and there were no ROM discontinuations due to AEs. AEs were reported in six patients (54.6%), without any grade ≥ 3 AEs. AEs included upper respiratory infection, muscle ache, joint ache, diarrhea, pruritus, and dizziness in one patient (9.1%), respectively.

### Relapse, clonal evolution, and survival

At a median follow-up of 8 months (range: 6–8 months), only 12.5% (1/8) of responders who maintained ROM for 3 months relapsed 5 months after stopping ROM at 3 months. The responder reached PR in 1 month but did not reach CR during the entire follow-up period. His highest hemoglobin and platelet levels were 135 g/L and 128 × 10^9^/L, respectively. While his hemoglobin remained normal throughout the follow-up, his platelet count dropped to 26 × 10^9^/L at 5 months. The patient refused to receive ROM again. None of the six patients who had once reached CR relapsed at the end of follow-up. One patient kept ROM at the maximum dosage until the end of follow-up and remained CR. One patient stopped ROM at 3 months and maintained CR until the end of follow up, with CR persisted without treatment for 3 months. After achieving CR, two patients tapered ROM to 10 μg/kg per week and then maintained PR at the end of follow-up. Two patients discontinued ROM at 2 and 3 months, and then maintained PR at the end of follow-up, both with CR persisted without treatment for 1 month. During follow-up, no deaths, new myelodysplastic syndrome, or PNH clonal evolution were observed. Chromosomal karyotyping was performed in three patients at the end of the follow-up, and no new chromosomal abnormalities were detected.

## Discussion

ROM has shown remarkable efficacy and safety in treating refractory AA. However, the optimal starting dosage of ROM and its efficacy and safety in patients with refractory AA who fail to respond to other types of TPO-RAs remain unclear. Our results demonstrated that high-dose ROM was an effective treatment option with a rapid response for patients with difficult AA, refractory to oral TPO-RAs. EPAG, hetrombopag, avatrombopag, and ROM have been successively launched in China. Before the availability of ROM, we attempted to use avatrombopag after the failure to respond to EPAG in patients with refractory AA and achieved some success [21–23]. However, some patients still failed to respond to these TPO-RAs. Notably, we found in our study that with an initial and maximal ROM dose of 20 μg/kg per week, 72.7% of patients reached PR in a median of 1 month (range: 1–3 months), 54.5% of patients reached CR in a median of 2.5 months (range: 1–3 months), and 27.3% of patients had a trilineage response. These results indicate that ROM may still be effective in patients with difficult AA who had failed to respond to IST and oral TPO-RAs.

ROM has laid the general foundation for the efficacy in patients with refractory AA, similar to other types of oral TPO-RAs [14]. Experiments *in vitro* and in mice have shown that the activation of the TPO receptor is especially crucial for platelet production and together with other growth factors can promote the maturation of myeloid and erythroid lineages[24–29]. However, EPAG, hetrombopag, and avatrombopag are small molecule non-peptides that bind to the TPO receptor’s transmembrane and juxta membrane domains [30]. ROM is a complex consisting of peptides and an IgG-Fc domain, which binds directly to the extra-cytoplasmic domain competitively with endogenous TPO and can remove TPO from its receptor. Furthermore, although Janus kinase 2 and signal transducer and activator of transcription 5 (JAK2/STAT5), STAT3, phosphatidylinositol 3-kinases and Protein Kinase B (PI3K/AKT), and extracellular-signal-regulated kinase (ERK) pathways were all down-stream pathways of TPO-RAs [30,31], only ROM stimulated megakaryocytes and hematopoietic stem cells through a stronger activation of the PI3K/AKT pathway, compared to the STAT and ERK pathways [32]. However, other TPO-RAs bound to the TPO receptor’s transmembrane and juxta membrane domain depended more on the STAT and AKT pathway [29,32,33]. Collectively, switching from TPO-RA to ROM could be encouraged if the patient showed no response to other types of TPO-RA.

Moreover, dosage also plays a significant role in efficacy. Previous studies have suggested that the pharmacodynamics of ROM are not linear and that dosage affects its pharmacodynamics [13,34]. This is consistent with our results. We chose the highest ROM dosage ever reported, 20 μg/kg per week as the initial and maximal dosage in most patients. Our study presented the most promising results compared with those of previous reports of lower dosages of ROM in patients with refractory AA who failed to respond to EPAG. The 3-month ORR of 72.7% of patients in our cohort was comparable with that of 76% of patients in the Japanese cohort [16] where patients with refractory AA received ROM at an initial dosage of 10 μg/kg per week and a median maximal dosage of approximately 20 μg/kg per week. This was also higher than the 12.5% of patients in the French cohort where patients with refractory SAA received ROM at an initial dosage of 5 μg/kg per week and a maximal dosage of approximately 10 μg/kg per week. The 9-week ORR of 72.7% of patients in our cohort was also a little higher than the 9-week ORR of 70% of patients [15] in the Korean cohort, in which patients with refractory SAA received ROM at an initial dosage of 10 μg/kg per week and a median maximal dosage of approximately 20 μg/kg per week. Similarly, the trilineage response rate in our cohort was 27.3%, slightly higher than the 19% response rate at 12 weeks in a Japanese cohort [16]. At 3 months, of the patients dependent on platelet transfusion, 54.5% became independent, and 57.1% of packed-red-cell-dependent patients became independent, which was higher than the previously reported 33% platelet and packed red cell independence rates at 3 months in a Japanese cohort [16]. In addition, compared with other studies with a lower dosage of ROM, our cohort demonstrated the most rapid response. The Korean cohort calculated the response rate at approximately 6–14 months, whereas the response was reached after a median of 1 month (range: 1–3 months) in our cohort. The first CR patient in the Japanese cohort reached CR at approximately 7 months [16], whereas all six patients who once achieved CR in our cohort reached CR within 3 months. The high effectiveness and rapid response in our cohort may be because the initial and maximal dosages were the highest, which could lead to consistent stimulation of hematopoietic stem and progenitor cells. In contrast to other studies, most of our patients had NSAA, and only one patient received ATG+CsA, whereas others received only CsA. Therefore, our patients may have had insufficient IST. The two patients with VSAA/SAA in our study had an ORR of 50%, similar to that reported in the Japanese cohort (55%) and greater than that in the French cohort (12.5%), indicating that dosage may be very crucial. In addition, all patients had received at least two types of TPO-RAs before ROM in our study, which may have contributed to the higher response of our patients in a late-onset response-exclusive manner. For instance, EPAG and hetrombopag are iron chelation agents [35–38] that may regulate hematopoietic stem cells and increase the sensitivity of patients with refractory AA to ROM by decreasing ferritin.

ROM was generally well-tolerated in this retrospective study, with no grade ≥ 3 AEs and discontinuation due to ROM-related AEs, which aligned with previous safety assessments. Three other cohorts with the largest dose of 20 μg/kg per week showed that ROM was well tolerated [16–18]. Kohei Hosokawa [16] reported no severe ROM-related AEs requiring discontinuation or dosage reduction with a maximal dosage of 20 μg/kg per week. Jun Ho Jang reported 3 grade ≥ 3 AEs (abnormal hepatic function, alanine aminotransferase increased, and γ-glutamyl transferase increased) among 31 patients with a maximal dosage of 20 μg/kg per week [17]. Kinuko Mitani reported one AE grade ≥ 3 (weight increase) among 27 patients with a maximal dosage of 20 μg/kg per week [18]. The low or no incidence of AEs in all these studies supported the safety of ROM at 20 μg/kg per week. No PNH clonal evolution, new karyotype abnormalities, or new myelodysplastic syndrome were observed in our study, which is consistent with previous reports [15,16]. Notably, given the median duration of the observation period in this study was 8 months (range: 6–8 months), it might not be sufficient to fully assess the risk of clonal evolution.

Moreover, before ROM was discontinued, no patient relapsed. Patients who achieved CR in our cohort maintained their response even after ROM was discontinued. These results were consistent with those of previous studies highlighting ROM’s low recurrence rate and maintenance of response to ROM even after discontinuation. A Japanese cohort with a ROM duration of 11 to 18 months reported no relapse [16]. Five patients experienced dose tapering at 53–157 weeks and maintained response for 53–490 days [15]. Previous studies have suggested that better T-regulatory function [39], decrease platelet antibodies through increased exposure to platelet autoantigens [40,41], and regeneration of the stromal population induced by the recovery of HSC [15] may contribute to the maintenance of response after ROM discontinuation. However, relapse had also been reported in patients with continuous ROM at the maximum dose of 20 μg/kg for more than 8 weeks, although this is very rare [18].

However, this study has some limitations. First, our study was retrospective, and the sample size was very small, which may limit the generalizability of our findings. Furthermore, our patients may have been highly selected because all had been treated with a full dose of IST and oral TPO-RAs. Second, the duration of ROM treatment and follow-up was short.

## Conclusion

In conclusion, our study provides valuable insights into the efficacy and safety of high-dose ROM with an initial dose of 20 μg/kg per week in patients with refractory AA, which merits further investigations.

## Data Availability

The datasets are not publicly available due to personal data protection reasons but are available from the corresponding author upon reasonable request.
